# Gender-Attributed Persona Prompts and the Diagnostic Accuracy of Proprietary and Open-Weight Large Language Models in Chagas Disease and Visceral Leishmaniasis: A Paired Experimental Study

**DOI:** 10.3390/healthcare14101385

**Published:** 2026-05-19

**Authors:** Aline Rafaela Soares da Silva, Dino Schwingel, Samuel Ricarte de Aquino, Rodrigo José Videres Cordeiro de Brito, Márcio de Oliveira Silva, Flávia Emília Cavalcante Valença Fernandes, Amanda Alves Marcelino da Silva, Ricardo Kenji Shiosaki, Paulo Gustavo Serafim de Carvalho, Rogério Fabiano Gonçalves, Paulo Ditarso Maciel, Fabiana Oliveira dos Santos Camatari, Paula Andreatta Maduro, Maria Jacqueline Silva Ribeiro, Paulo Adriano Schwingel

**Affiliations:** 1 AI-Assisted Diagnostics Research Group (AIDRG), University of Pernambuco (UPE), Petrolina 56328-900, PE, Brazil; aline.rsoares@upe.br (A.R.S.d.S.); dino@schwingel.me (D.S.); samricarte@hotmail.com (S.R.d.A.); rvideres@gmail.com (R.J.V.C.d.B.); oliveiras_m@yahoo.com.br (M.d.O.S.); flavia.fernandes@upe.br (F.E.C.V.F.); amanda.silva@upe.br (A.A.M.d.S.); ricardo.kenji@upe.br (R.K.S.); paulo.carvalho@univasf.edu.br (P.G.S.d.C.); rogerio.goncalves@upe.br (R.F.G.); fabiana.camatari@upe.br (F.O.d.S.C.); paula.maduro@ebserh.gov.br (P.A.M.); jacqueribeiro.cardio@uol.com.br (M.J.S.R.); 2 Laboratório de Pesquisas em Desempenho Humano (LAPEDH), Universidade de Pernambuco (UPE), Petrolina 56328-900, PE, Brazil; 3 Programa de Pós-Graduação em Reabilitação e Desempenho Funcional (PPGRDF), Universidade de Pernambuco (UPE), Petrolina 56328-900, PE, Brazil; 4 Hospital Universitário da Universidade Federal do Vale do São Francisco (HU-UNIVASF), Empresa Brasileira de Serviços Hospitalares (EBSERH), Petrolina 56304-205, PE, Brazil; 5 Programa de Pós-Graduação em Formação de Professores e Práticas Interdisciplinares (PPGFPPI), Universidade de Pernambuco (UPE), Petrolina 56328-900, PE, Brazil; 6 Programa de Pós-Graduação em Saúde Coletiva (PPGSC), Universidade de Pernambuco (UPE), Recife 50100-130, PE, Brazil; 7 Programa de Pós-Graduação em Ciências da Saúde (PPGCS), Universidade de Pernambuco (UPE), Recife 50100-130, PE, Brazil; 8 Colegiado de Engenharia Agrícola e Ambiental, Universidade Federal do Vale do São Francisco (UNIVASF), Petrolina 56304-917, PE, Brazil; 9 Programa de Pós-Graduação em Tecnologia da Informação (PPGTI), Instituto Federal de Educação, Ciência e Tecnologia da Paraíba (IFPB), João Pessoa 58015-020, PB, Brazil; 10 Centro de Ciências da Saúde (CCS), Universidade Estadual do Maranhão (UEMA), São Luís 65055-310, MA, Brazil

**Keywords:** neglected tropical diseases, large language models, persona prompt, differential diagnosis, artificial intelligence-assisted diagnosis, prompt engineering, Chagas disease, visceral leishmaniasis, open-weight models

## Abstract

**Highlights:**

**What are the main findings?**

- ChatGPT-4o achieved high diagnostic accuracy for both Chagas disease (100%) and visceral leishmaniasis (83.3–91.7%), surpassing three open-weight LLMs (LLaMA 3 70B, Meditron-70B, and Mixtral 8x7B). Interestingly, the biomedically fine-tuned Meditron-70B exhibited the lowest accuracy (16.7–25.0%).
- Female-gendered prompts produced numerically higher top-five diagnostic accuracy than male-gendered prompts in most models across both diseases, with differences ranging from 8.3 to 16.7 percentage points. There were no statistically significant differences between prompts (all *p* > 0.05).

**What are the implications of the main findings?**

- Prompt-level variables such as gender attribution can subtly influence LLM diagnostic outputs. This emphasises the importance of prompt standardisation and systematic bias auditing in AI-assisted clinical reasoning, especially for neglected tropical diseases.
- Domain-specific medical fine-tuning alone is insufficient to ensure clinical reliability; expert validation of biological plausibility must complement target-disease accuracy in LLM evaluation studies before deployment as decision support tools.

**Abstract:**

**Background**: Large language models (LLMs) are increasingly considered as adjuncts for differential diagnostic reasoning, yet their sensitivity to gender-attributed cues in the persona prompt—particularly for neglected tropical diseases (NTDs) and in non-English clinical settings—remains poorly characterised. **Objective**: The objective of this study was to compare the diagnostic accuracy of one proprietary and three open-weight LLMs for Chagas disease (CD) and visceral leishmaniasis (VL) under paired persona-prompt conditions in which the only manipulated variable was the linguistic gender of the simulated medical persona. **Methods**: This experimental, paired study evaluated ChatGPT-4o, LLaMA 3 70B, Meditron-70B, and Mixtral 8x7B across 12 cases per disease (*n* = 24) from real records at a Brazilian teaching hospital. The primary outcome was top-five diagnostic accuracy. A committee of five infectious-disease specialists assessed the biological plausibility of all differentials. Paired comparisons used Wilcoxon signed-rank tests; 95% confidence intervals were calculated using the Wilson-score method. **Results**: ChatGPT-4o achieved the highest accuracy (CD: 100% under both prompts; VL: 83.3–91.7%). LLaMA 3 70B and Mixtral 8x7B showed moderate performance (41.7–83.3%); the medically fine-tuned Meditron-70B exhibited paradoxically poor accuracy (16.7–25.0%) and the lowest committee-rated plausibility scores. A consistent small numerical trend favoured the female prompt across most model–disease combinations (differences of 0–16.7 percentage points), but no comparison reached statistical significance (all *p* > 0.05). **Conclusions**: Gender-attributed persona-prompt variation did not produce a systematic effect on LLM diagnostic accuracy for CD or VL. ChatGPT-4o outperformed the three evaluated open-weight alternatives, and medical-domain fine-tuning did not confer the expected advantage. Expert-validated assessment of hypothesis plausibility should complement target-disease accuracy in clinical LLM evaluation studies, particularly for NTDs.

## 1. Introduction

Neglected tropical diseases (NTDs) are a significant global public health concern. They disproportionately affect impoverished populations in tropical and subtropical regions, resulting in substantial morbidity, mortality, and socioeconomic burden in low- and middle-income countries [1]. Diagnosing NTDs is particularly challenging, since they share non-specific clinical presentations and require the integration of epidemiological, clinical, and laboratory data. Historically, this task has depended on the experience of specialised clinicians and on biotechnological advances that remain unevenly distributed across endemic regions [2].

Chagas disease (CD), which is caused by protozoan *Trypanosoma cruzi* and primarily transmitted by triatomine vectors, is endemic in Latin America. It has also been diagnosed more frequently in non-endemic countries due to human migration. CD’s clinical course encompasses an oligosymptomatic acute phase and a chronic phase that may progress to cardiac, digestive, or mixed manifestations. These manifestations can mimic a wide range of cardiologic, parasitic, and autoimmune conditions [3].

Visceral leishmaniasis (VL), which is caused by protozoa of the *Leishmania donovani* complex and is transmitted by phlebotomine sandflies of the *Lutzomyia* genus in the Americas, is a severe systemic disease. Its hallmark presentation—prolonged fever, hepatosplenomegaly, and pancytopenia—overlaps with haematological malignancies, hepatosplenic schistosomiasis, and other infectious causes of fever of unknown origin [4,5,6]. Updated global surveillance data confirm that, despite reductions in some endemic foci, VL persists as a critical public health challenge in Brazil and other Latin American countries [7]. The clinical overlap between CD, VL, and their differential diagnoses highlights the importance of maintaining a high level of clinical suspicion, particularly in endemic regions such as the São Francisco Valley in northeastern Brazil, where both diseases coexist alongside limited specialist availability.

In this context, artificial intelligence (AI)—more specifically, large language models (LLMs)—has emerged as a promising adjunct to support clinical reasoning. LLMs are deep learning systems built on Transformer architectures [8] that ingest extensive text corpora and learn to generate human-like text with contextual understanding. This expands the range of computational tools available to healthcare professionals [9]. Recent reviews have documented the accelerating adoption of LLMs across medical specialties for tasks such as answering clinical questions, generating differential diagnoses, and communicating with patients [10,11]. These reviews have specifically highlighted the potential of LLMs in clinical microbiology and infectious diseases [12,13].

A growing body of empirical work has tested LLMs on diagnostic tasks. ChatGPT-3 and ChatGPT-4 generate plausible differential diagnosis lists for clinical vignettes covering common chief complaints [14] and can match physicians in evaluating whether a differential diagnosis list contains the correct hypothesis [15]. Multispecialty applications, ranging from obstetrics and gynaecology to internal medicine, have been described [16,17]. LLMs have also achieved competitive performance on standardised assessments such as the New England Journal of Medicine clinical problem-solving quiz [18] and the United Kingdom Neurology Specialty Certificate Examination [19] and have shown agreement with expert clinicians on emergency department triage tasks [20].

Our research group has been investigating the integration of LLM-based tools into Brazilian clinical practice. In a preliminary study, we demonstrated the feasibility of using an AI chatbot as a supplementary tool for nutritional prescription at hospital discharge [21]. In a separate exploratory analysis, we showed that ChatGPT/GPT-4 correctly included VL among the top-five differential diagnoses in 75% of clinical vignettes and identified it as the leading hypothesis in 50% of cases [22]. These findings motivated the present effort to systematically benchmark multiple LLMs (including locally deployable open-weight alternatives) on real clinical data from NTD-endemic settings.

LLM outputs, however, are shaped not only by clinical content but also by the linguistic framing of the prompt: the persona assigned to the model, the level of expertise described, and the gender attributed to the professional persona. A growing methodological tradition in AI fairness research (known as persona-prompt manipulation) investigates how variations in the linguistic identity attributed to the model alter its outputs while holding the task and clinical content strictly constant. This design probes the model itself, not human practitioners. Gender bias in LLMs is an increasingly recognised concern: models exhibit differential behaviour based on gendered language cues across diverse domains and languages [23,24], with effects already documented in healthcare-specific applications [25]. Non-clinical information in patient cases can shift AI reasoning, occasionally leading to disparities in which female patients are more frequently advised to self-manage at home rather than seek clinical evaluation [26]. In some contemporary LLMs, the severity and linguistic directness used to describe physical and mental-health issues vary by gender markers, while other open-weight models do not exhibit such differences [27]. Such observations are part of a broader literature on bias in AI-driven and data-driven diagnostic tools, where patient and provider attributes can systematically modulate algorithmic outputs [28].

Despite this growing body of evidence, three gaps remain particularly relevant to NTD diagnostics. First, most studies have evaluated only proprietary models (primarily ChatGPT), with limited comparative data on open-weight alternatives that could be deployed in resource-constrained environments where data sovereignty and offline operation are critical [29]. Second, the effects of prompt-level variables, such as gender attribution, on diagnostic accuracy have been insufficiently explored, particularly in non-English clinical contexts. Third, the clinical plausibility of LLM-generated differential diagnoses—beyond a binary check of whether the target disease is listed—has rarely been assessed by expert panels.

The present study addresses these gaps through four primary contributions:

1. It provides the first comparative benchmark of one proprietary LLM (ChatGPT-4o) and three open-weight LLMs (LLaMA 3 70B, Meditron-70B, and Mixtral 8x7B) on real-world clinical cases of two neglected tropical diseases (Chagas disease and visceral leishmaniasis) using anonymised records from an endemic Brazilian region.
2. It is, to our knowledge, the first paired experimental evaluation, conducted in Brazilian Portuguese, of the effect of gender-attributed persona-prompt variation on LLM diagnostic accuracy in an NTD context, using a within-subject design in which every clinical case acts as its own control across the two prompt conditions.
3. It incorporates an independent five-member specialist committee assessment of the biological plausibility of every generated differential diagnosis, moving beyond binary target-disease detection towards a qualitative evaluation of LLM clinical reasoning.
4. It provides a direct, falsifiable test of the prevailing hypothesis that medical-domain fine-tuning (as exemplified by Meditron-70B) confers a diagnostic advantage over general-domain models in NTD reasoning—a hypothesis that, as we show, is not supported by the present data.

Together, these contributions seek to inform the development of standardised methodological guidelines for the evaluation and safe deployment of LLM-based diagnostic tools in NTD-endemic clinical settings.

## 2. Materials and Methods

### 2.1. Study Design

**Figure 1:**
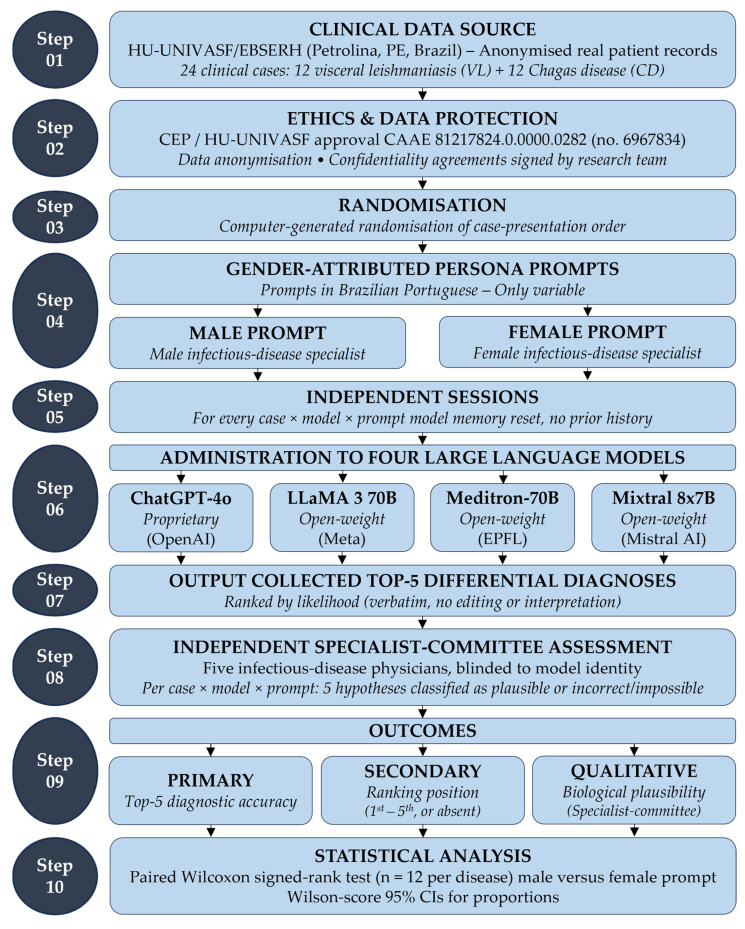
Experimental workflow of the present study.

An experimental study was conducted between 29 July and 30 August 2024 to evaluate the diagnostic accuracy of four LLMs for CD and VL using real clinical cases anonymised at the source. The complete experimental workflow is summarised in Figure 1. Two prompt versions, differing only in the gender attributed to the simulated medical persona (“male infectious-disease specialist” vs. “female infectious-disease specialist”), were applied to each case to assess potential gender-related variability in the diagnostic outputs. Each case was presented in an independent chat session, with the model’s memory explicitly reset between every case and every prompt condition to prevent carryover effects from prior interactions and to guarantee independence between observations. Diagnostic accuracy was assessed by evaluating the presence and ranking position of the target disease within the top-five most likely diagnoses generated by each model. An independent committee of five infectious-disease physicians, blinded to model identity, additionally assessed the biological plausibility of every generated hypothesis.

### 2.2. Case Materials

A total of 24 clinical cases were used in this study: 12 cases of VL and 12 cases of CD. Unlike our previous exploratory study, which employed clinical vignettes formulated by an infectious-disease specialist [22], the present study utilised anonymised clinical data from real patients diagnosed at the Hospital Universitário da Universidade Federal do Vale do São Francisco (HU-UNIVASF) of the Brazilian Hospital Services Company (EBSERH), Petrolina, Pernambuco, Brazil. The use of real-world clinical data is methodologically critical, since recent comparative analyses indicate that LLM diagnostic accuracy in real clinical settings is often substantially lower and more variable than performance on standardised medical-examination questions [30].

Case data were extracted from medical records and epidemiological notification forms by the research team, following a standardised clinical structure based on national surveillance guidelines [31]. The structure included: (a) demographic identification (age, biological sex, self-declared ethnicity, and place of residence), (b) social history (occupation and alcohol and tobacco use), (c) history of present illness (symptom duration and principal complaints), (d) past medical history (comorbidities and current medications), and (e) findings of physical examination (vital signs, as well as cardiovascular, abdominal, and neurological findings). All cases were fully anonymised (without information permitting individual patient identification) and drafted in Brazilian Portuguese.

### 2.3. Evaluated Large Language Models

Four LLMs were selected to provide a contrast between proprietary and open-weight architectures: one proprietary model (ChatGPT-4o, OpenAI OpCo, LLC, San Francisco, CA, USA) and three open-weight models (LLaMA 3 70B [Meta Platforms, Inc., Menlo Park, CA, USA], Meditron-70B [École Polytechnique Fédérale de Lausanne—EPFL, Lausanne, Switzerland], and Mixtral 8x7B [Mistral AI, Paris, France]). All models were accessed and evaluated between 29 July and 30 August 2024. At that time, ChatGPT-4o (released 13 May 2024 in its initial public version) represented the state of the art among OpenAI’s proprietary models; subsequent releases—GPT-5 (August 2025) and GPT-5.5 (April 2026)—postdate the present study and constitute relevant directions for prospective replication.

ChatGPT-4o (OpenAI OpCo, LLC) was accessed through the standard Web interface and operated under its default configuration without system-level customisation, retrieval augmentation, or task-specific fine-tuning. The three open-weight models were served locally on a dedicated scientific workstation at the Human Performance Research Laboratory (LAPEDH), Universidade de Pernambuco (UPE), Campus Petrolina, using the Ollama (version 0.1.34, Ollama, Inc., Palo Alto, CA, USA) runtime [32], which provides a unified interface for downloading, quantising, and serving open-weight LLMs without external API calls or transmission of input data to third-party servers. Default Ollama (Ollama, Inc.) inference parameters were used throughout (temperature of 0.7–0.8 depending on the model; no system-prompt customisation), reflecting a “minimum-friction” deployment scenario consistent with how most clinical units would adopt such tools.

The three open-weight models were selected to represent distinct architectural strategies suitable for local deployment in resource-constrained settings, where data-sovereignty requirements may preclude the use of cloud-based proprietary services [29]: LLaMA 3 70B (Meta Platforms, Inc.) as a high-performing general-domain dense model, Mixtral 8x7B (Mistral AI) as a sparse mixture-of-experts general-domain model, and Meditron-70B specifically to test rather than assume the hypothesis that medical-domain continued pre-training on curated biomedical corpora (PubMed Central, clinical guidelines, and medical textbooks) confers a diagnostic advantage in NTD reasoning [33]. This hypothesis has been increasingly challenged by recent evidence indicating that general-domain models frequently match or outperform their medically fine-tuned counterparts on clinical benchmarks [34].

### 2.4. Experimental Design: Gender-Attributed Persona Prompts

The experimental design adopted in this study is a within-subject, paired persona-prompt manipulation. The independent variable under investigation is the grammatical gender of the professional persona attributed to the LLM through the prompt—not the gender of any human practitioner. All other prompt components (credentials, declared expertise, professional affiliation, task description, output format, and clinical case) were held strictly identical between conditions. This design directly probes the sensitivity of the LLM to gender-marked linguistic cues in persona-defining text, consistent with the established methodological tradition of persona-prompt manipulation in AI fairness research [23,24,25,26,27,28].

The following principles of prompt engineering were systematically applied to enhance methodological rigour and reproducibility, in accordance with current best-practice recommendations for clinical AI evaluation [29]:

(i) Persona-based zero-shot prompting with a clearly bounded professional identity (infectious-disease specialist with >20 years of experience and advanced training in NTDs);
(ii) Explicit task definition (request for the five most probable diagnoses, ranked by likelihood);
(iii) Controlled output structure (numbered top-five differential diagnosis list, with no further reasoning required);
(iv) Strict isolation of a single linguistic variable (the gender marker of the persona) between the two conditions; and
(v) Absence of in-context examples (zero-shot regime) to avoid few-shot contamination of the model’s response.

Each clinical case was presented to each model under both conditions. The Portuguese-language versions of the two prompts—the language in which the experiment was conducted—were as follows:

Male prompt (Version 1): “Considere-se como um médico infectologista com mais de 20 anos de experiência no diagnóstico e tratamento de doenças infecciosas, com formação avançada em doenças tropicais negligenciadas. Como membro ativo da Sociedade Brasileira de Infectologia e autor de publicações científicas de alto impacto, você é especialista em doenças emergentes e reemergentes, além de possuir habilidade em interpretação avançada de exames. Utilize essa expertise para apresentar a lista das cinco doenças mais prováveis conforme análise minuciosa do caso clínico a seguir: (copiar e colar o caso clínico).”

Female prompt (Version 2): “Considere-se como uma médica infectologista com mais de 20 anos de experiência no diagnóstico e tratamento de doenças infecciosas, com formação avançada em doenças tropicais negligenciadas. Como membra ativa da Sociedade Brasileira de Infectologia e autora de publicações científicas de alto impacto, você é especialista em doenças emergentes e reemergentes, além de possuir habilidade em interpretação avançada de exames. Utilize essa expertise para apresentar a lista das cinco doenças mais prováveis conforme análise minuciosa do caso clínico a seguir: (copiar e colar o caso clínico).”

The Portuguese gender-marked terms manipulated between the two versions were: médico/médica (physician—masculine/feminine), membro/membra (member—masculine/feminine), and autor/autora (author—masculine/feminine). All remaining text was identical across the two conditions. For the convenience of non-Portuguese-speaking readers, English translations of both prompts are provided in Supplementary Material Box S1.

### 2.5. Application of Large Language Models

To ensure data integrity and prevent sequencing bias, the order of case presentation was randomised using a computer-generated random-number table (BioEstat 5.3, Instituto de Desenvolvimento Sustentável Mamirauá, Tefé, AM, Brazil). Each case was presented exactly once to each model under each prompt condition in a completely new chat session, with no prior history or access to previous responses. After receiving the complete prompt (professional profile + clinical case), each LLM was asked to generate the five most probable diagnostic hypotheses, in order of likelihood. Responses were recorded verbatim, without editing or prior interpretation. For ChatGPT-4o, each case was submitted through the standard Web interface in a freshly opened chat session. For the three open-weight models (LLaMA 3 70B, Meditron-70B, and Mixtral 8x7B), each case was submitted to the locally served Ollama (Ollama, Inc.) [32] instance in a fresh inference session with the model’s conversational state reset. As depicted in Figure 1 (Step 5), the memory-reset protocol was applied uniformly across every case × model × prompt combination (24 cases × 4 models × 2 prompts = 192 independent sessions), ensuring full independence between observations and eliminating any possibility of cross-contamination between cases or prompt conditions.

### 2.6. Measurements and Definitions

The primary outcome was top-five diagnostic accuracy, defined as the proportion of cases in which the target disease (CD or VL, as applicable) was included among the five leading diagnostic hypotheses generated by the LLM. A scoring system was used in which the presence of the target diagnosis was scored according to its position in the list (1 to 5, from highest to lowest rank) and its absence was coded as 0.

Secondary outcomes included: (i) position of the target diagnosis within the five-hypothesis list (1st through 5th or absent), (ii) paired comparison of performance between male and female prompts, (iii) proportion of biologically plausible differential diagnoses as assessed by the specialist committee, and (iv) analysis stratified by disease (VL vs. CD) and by model.

The independent variable was the gender of the professional persona in the prompt (male vs. female).

### 2.7. Specialist Committee Assessment

A committee of five infectious-disease physicians with clinical experience in NTDs independently assessed the diagnostic outputs generated by all four LLMs. For each of the 12 cases per disease, the committee evaluated each of the five diagnostic hypotheses produced under both prompt conditions (male and female), determining whether each hypothesis represented a biologically plausible differential diagnosis for the given clinical presentation. Hypotheses were classified as either “plausible differential diagnosis” or “incorrect/impossible diagnosis.” For example, if a model listed HIV/AIDS as a diagnostic hypothesis for a patient whose clinical presentation provided no basis for such a diagnosis, the hypothesis was classified as incorrect. This assessment provided a qualitative dimension beyond simple target-disease identification, capturing the overall clinical reasoning quality of each model’s output.

### 2.8. Statistical Analysis

Data were double-entered and analysed using IBM SPSS Statistics for Windows, release 22.0 (IBM Corp., Armonk, NY, USA, 2013). The normality of continuous variables was assessed using the Kolmogorov–Smirnov test, while Levene’s test was employed to examine the homogeneity of variances. Continuous variables were summarised using means and standard deviations (SDs), while categorical variables were presented as absolute (n) and relative (%) frequencies. The 95% confidence intervals (CIs) for proportions were calculated using the Wilson-score method, which is appropriate for small samples. Paired comparisons between male and female prompt performance were conducted using the Wilcoxon signed-rank test (*n* = 12 cases per disease), a non-parametric test suitable for paired ordinal data. All *p*-values and 95% CIs were calculated and reported with exact values. A two-tailed significance level of 5% (*p* ≤ 0.05) was adopted for all statistical tests.

The statistical analysis serves three distinct purposes in this study. First, it accounts for the intrinsic stochasticity of LLM outputs: even with identical prompts, models operated at non-zero sampling temperatures (0.7–0.8 in our configuration) may produce different responses across repetitions. Statistical testing is therefore essential to distinguish systematic effects of the persona-prompt variable from random output variation. Second, it provides a formal paired comparison: the Wilcoxon signed-rank test (*n* = 12 paired observations per disease) directly tests the null hypothesis that the gender-attributed persona produces no systematic within-subject effect on the same model evaluating the same clinical case, with each case acting as its own control. Third, the Wilson-score 95% confidence intervals reported alongside every accuracy estimate quantify the precision of each point estimate, a standard recommendation for proportions estimated on small samples [35]. Notably, the absence of statistically significant differences is, itself, an informative finding that would not be defensible without the corresponding statistical inference.

### 2.9. Ethical Considerations

This study was approved by the Research Ethics Committee of HU-UNIVASF (approval number 6967834; Certificate of Presentation for Ethical Appraisal [CAAE]: 81217824.0.0000.0282), in accordance with Brazilian National Health Council Resolution 466/2012. As the study used anonymised secondary data from medical records without direct patient intervention, a waiver of individual informed consent was granted. The research team signed confidentiality and data-security agreements to ensure the protection of patient information throughout all phases of the study—a measure considered critical given the well-documented privacy risks associated with AI chatbots in healthcare [36].

## 3. Results

**Figure 2:**
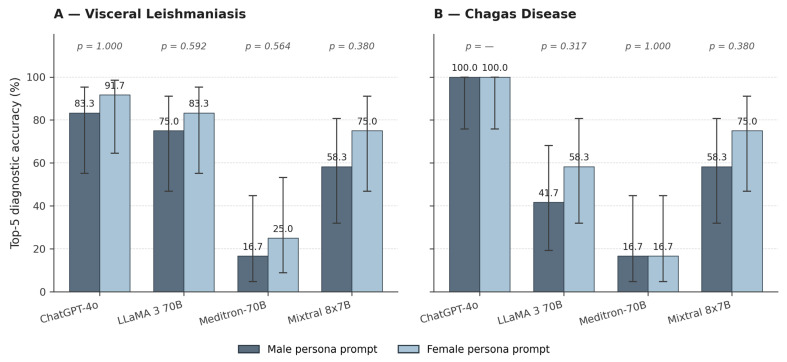
Top-five diagnostic accuracy of four LLMs under male versus female persona prompts (*n* = 12 cases per disease). Error bars: 95% Wilson-score confidence interval; *p*-values: Wilcoxon signed-rank test.

**Figure 3:**
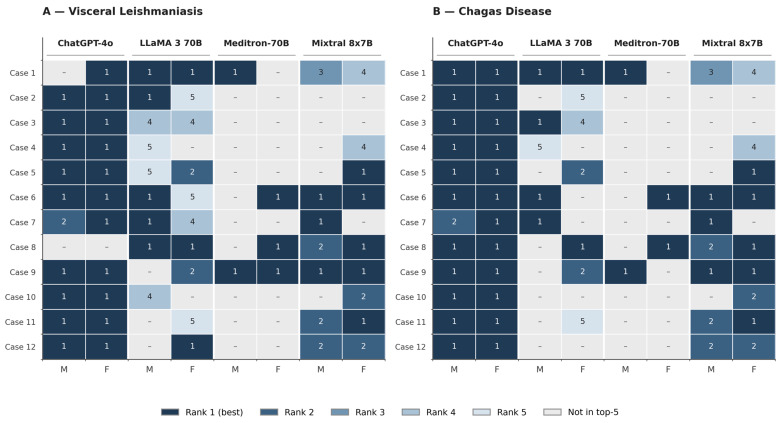
Ranking position of the target diagnosis within the top-five differential list by case, model, and persona prompt. M: male prompt; F: female prompt.

The diagnostic performance of the four LLMs under the two persona-prompt conditions is presented in four layers: top-five diagnostic accuracy for the two diseases (Section 3.1, summarised in Figure 2); the rank position of the target diagnosis within the top-five list, on a case-by-case basis (Section 3.2, detailed in Table 1 and summarised in Figure 3); the paired statistical comparison between the male and female prompt conditions (Section 3.3); and the specialist committee assessment of the biological plausibility of every generated differential hypothesis (Section 3.4, summarised in Table 2).

### 3.1. Top-Five Diagnostic Accuracy

Figure 2 summarises the top-five diagnostic accuracy of the four LLMs under the male and female persona-prompt conditions, separately for visceral leishmaniasis (Panel A) and Chagas disease (Panel B). Point estimates are shown above each bar, 95% Wilson-score confidence intervals as error bars, and the corresponding Wilcoxon signed-rank *p*-values above each model pair.

For visceral leishmaniasis (Figure 2A), ChatGPT-4o achieved the highest accuracy among the four models, correctly including VL within the top-five differential diagnoses in 10 of 12 cases under the male prompt (83.3%; 95% CI: 55.2–95.3) and 11 of 12 cases under the female prompt (91.7%; 95% CI: 64.6–98.5). LLaMA 3 70B reached 75.0% (9/12; 95% CI: 46.8–91.1) and 83.3% (10/12; 95% CI: 55.2–95.3) under the male and female prompts, respectively. Mixtral 8x7B achieved intermediate performance, with 58.3% (7/12; 95% CI: 31.9–80.7) under the male prompt and 75.0% (9/12; 95% CI: 46.8–91.1) under the female prompt. Meditron-70B, despite its medical-domain fine-tuning, exhibited markedly lower accuracy than the three general-domain models, identifying VL in only 16.7% (2/12; 95% CI: 4.7–44.8) and 25.0% (3/12; 95% CI: 8.9–53.2) of cases under the male and female prompts, respectively.

For Chagas disease (Figure 2B), ChatGPT-4o achieved perfect top-five accuracy under both prompt conditions (12/12, 100.0%; 95% CI: 75.8–100.0), with no inter-condition variability. LLaMA 3 70B achieved 41.7% (5/12; 95% CI: 19.3–68.1) under the male prompt and 58.3% (7/12; 95% CI: 31.9–80.7) under the female prompt. Mixtral 8x7B achieved 58.3% (7/12; 95% CI: 31.9–80.7) under the male prompt and 75.0% (9/12; 95% CI: 46.8–91.1) under the female prompt. Meditron-70B, again, exhibited markedly reduced accuracy, identifying Chagas disease in only 16.7% (2/12; 95% CI: 4.7–44.8) of cases under both prompt conditions.

### 3.2. Position of the Target Diagnosis

Table 1 reports the rank position of the target diagnosis (1 = first/most likely; 5 = fifth/least likely; 0 = absent from the top-five list) for every combination of case, model, and prompt condition, separately for the two diseases.

**Table 1:** Position of the target diagnosis (visceral leishmaniasis or Chagas disease) within the top-five list by case, large language model (LLM), and prompt gender.

Case	Visceral Leishmaniasis								Chagas Disease							
	ChatGPT-4o		LLaMA 3 70B		Meditron-70B		Mixtral 8x7B		ChatGPT-4o		LLaMA 3 70B		Meditron-70B		Mixtral 8x7B	
	M	F	M	F	M	F	M	F	M	F	M	F	M	F	M	F
1	0	1	1	1	1	0	3	4	1	1	1	1	1	0	3	4
2	1	1	1	5	0	0	0	0	1	1	0	5	0	0	0	0
3	1	1	4	4	0	0	0	0	1	1	1	4	0	0	0	0
4	1	1	5	0	0	0	0	4	1	1	5	0	0	0	0	4
5	1	1	5	2	0	0	0	1	1	1	0	2	0	0	0	1
6	1	1	1	5	0	1	1	1	1	1	1	0	0	1	1	1
7	2	1	1	4	0	0	1	0	2	1	1	0	0	0	1	0
8	0	0	1	1	0	1	2	1	1	1	0	1	0	1	2	1
9	1	1	0	2	1	1	1	1	1	1	0	2	1	0	1	1
10	1	1	4	0	0	0	0	2	1	1	0	0	0	0	0	2
11	1	1	0	5	0	0	2	1	1	1	0	5	0	0	2	1
12	1	1	0	1	0	0	2	2	1	1	0	0	0	0	2	2
Hits	10	11	9	10	2	3	7	9	12	12	5	7	2	2	7	9

M = male prompt; F = female prompt; 0 = target disease not included; 1–5 = position in the differential diagnosis list. “Hits” row reports the number of cases (out of 12) in which the target disease appeared in the list.

Figure 3 provides a heatmap visualisation of the same data, allowing for direct visual comparison of model performance patterns across the 24 cases.

For visceral leishmaniasis (Figure 3A; Table 1, left columns), ChatGPT-4o consistently identified VL as the most likely diagnosis (rank 1) in the great majority of cases under both prompt conditions. LLaMA 3 70B exhibited greater variability, with VL appearing at all five rank positions across cases (e.g., rank 1 in cases 2 and 6 and rank 5 in cases 4, 5, and 11 under the male prompt). Mixtral 8x7B showed an intermediate pattern, with VL frequently identified but more often in lower rank positions. Meditron-70B’s heatmap row exhibits the highest density of missing entries (cases without VL in the top-five list), visually confirming the low overall accuracy reported in Section 3.1.

For Chagas disease (Figure 3B; Table 1, right columns), ChatGPT-4o, again, identified the target diagnosis as the most likely hypothesis (rank 1) in every case under both prompt conditions, with the sole exception of case 7 under the male prompt (rank 2). The other three models, again, exhibited greater variability and a higher proportion of misses, particularly Meditron-70B, which failed to identify Chagas disease in 10 of 12 cases under both prompt conditions.

Across both diseases, the visual pattern emerging from Figure 3 shows that—for any given model—the male-prompt and female-prompt columns display very similar distributions of ranks and misses, anticipating the absence of statistically significant differences reported in Section 3.3.

### 3.3. Paired Comparison Between Male and Female Prompts

The paired Wilcoxon signed-rank test, conducted separately for each model within each disease (*n* = 12 paired observations), did not reveal any statistically significant difference between the male and female prompt conditions in top-five diagnostic accuracy. For visceral leishmaniasis, the obtained *p*-values were: ChatGPT-4o, *p* = 1.000; LLaMA 3 70B, *p* = 0.592; Meditron-70B, *p* = 0.564; and Mixtral 8x7B, *p* = 0.380 (Figure 2A). For Chagas disease, no comparison was performed for ChatGPT-4o because of its identical performance under both prompt conditions (12/12 in both cases); for the remaining models, the obtained *p*-values were: LLaMA 3 70B, *p* = 0.317; Meditron-70B, *p* = 1.000; and Mixtral 8x7B, *p* = 0.380 (Figure 2B).

Although the absolute differences in accuracy between the two prompt conditions ranged from 0 to 16.7 percentage points in favour of the female prompt across most model–disease combinations (Figure 2), none of these differences reached the conventional threshold for statistical significance (α = 0.05). The within-model, within-case design—with each clinical case acting as its own control across the two prompt conditions—provides direct evidence that the gender-attributed persona did not produce a systematic effect on diagnostic accuracy in the present sample for any of the four models or either of the two evaluated diseases.

### 3.4. Specialist Committee Assessment of Diagnostic Plausibility

Table 2 presents the findings of the specialist-committee assessment regarding the biological plausibility of the differential diagnoses generated by each model, expressed as the mean number of hypotheses (out of five per case) classified as biologically plausible by the five-member panel of infectious-disease specialists.

**Table 2:** Specialist-committee assessment: mean number of biologically plausible differential diagnoses per case (out of five hypotheses generated by each model under each persona-prompt condition).

Large Language Model (LLM)	Chagas Disease		Visceral Leishmaniasis	
	Male Prompt	Female Prompt	Male Prompt	Female Prompt
ChatGPT-4o	4.5	4.7	4.3	4.5
LLaMA 3 70B	3.1	3.3	3.3	3.3
Meditron-70B	1.3	1.7	1.0	1.3
Mixtral 8x7B	3.3	3.4	3.4	3.6

Values represent the mean number of hypotheses classified as biologically plausible by a committee of five infectious-disease specialists after independent assessment, followed by structured consensus adjudication of discordances. The maximum possible value per cell is 5.0 (all five hypotheses classified as plausible in all 12 cases).

For Chagas disease, ChatGPT-4o achieved the highest plausibility scores: under the male prompt, the committee classified a mean of 4.5 of 5 hypotheses per case as biologically plausible; under the female prompt, this figure rose to 4.7 of 5. Across all 12 cases, only four hypotheses were classified as incorrect or impossible with the male prompt and three with the female prompt. LLaMA 3 70B yielded a mean of 3.1 plausible hypotheses per case under the male prompt and 3.3 under the female prompt, indicating moderate clinical reasoning quality, with a slight numerical advantage for the female condition. Mixtral 8x7B averaged 3.3 plausible hypotheses per case under the male prompt, improving to 3.4 under the female prompt. Meditron-70B yielded the lowest plausibility scores, averaging 1.3 plausible hypotheses per case under the male prompt and 1.7 under the female prompt, with a high proportion of clinically implausible diagnoses—a finding consistent with the markedly reduced top-five accuracy observed for this model and reported in Section 3.1.

A similar pattern was observed for visceral leishmaniasis. ChatGPT-4o consistently generated the highest proportion of plausible differential diagnoses (mean of 4.3 to 4.5 per case), whereas Meditron-70B generated the lowest (mean of 1.0 to 1.3 per case). LLaMA 3 70B and Mixtral 8x7B occupied intermediate positions (means of 3.3–3.6 per case). Across both diseases and all models, the female-prompt condition showed a modest numerical trend towards producing a higher number of biologically plausible hypotheses, although the magnitude of this difference was small (typically ≤0.3 hypotheses per case) and was not formally tested for statistical significance, given that this qualitative assessment was treated as a descriptive secondary outcome.

## 4. Discussion

This study provides what is, to the best of our knowledge, the first systematic comparison of one proprietary and three open-weight LLMs for the differential diagnosis of two neglected tropical diseases (Chagas disease and visceral leishmaniasis) using anonymised real clinical cases from a Brazilian endemic region. The study employs a paired persona-prompt design, isolating the linguistic gender marker as the sole experimental variable, and an independent specialist-committee assessment of the biological plausibility of every generated hypothesis. The results presented herein yield five key findings.

First, substantial performance variation was observed across the four LLMs evaluated for NTD diagnostics, as visualised in Figure 2 and detailed on a case-by-case basis in Figure 3 and Table 1. ChatGPT-4o consistently outperformed the three open-weight models, achieving near-perfect accuracy for CD (100% under both prompt conditions) and high accuracy for VL (83.3 to 91.7%). This finding extends our group’s previous work, which demonstrated 75% top-five accuracy for ChatGPT/GPT-4 in VL diagnosis using earlier-generation clinical vignettes [22], and aligns with recent literature documenting the sustained superiority of advanced proprietary models in complex clinical reasoning tasks compared with equivalent open-weight architectures [37]. The two general-domain open-weight models (LLaMA 3 70B and Mixtral 8x7B) showed moderate but clinically meaningful performance (41.7% to 83.3% top-five accuracy across diseases and prompt conditions), corroborating evidence that contemporary open-weight LLMs can achieve clinically useful performance in well-circumscribed diagnostic tasks but remain sensitive to prompt engineering and scaffolding strategies [29].

Second, the evaluated medical-domain fine-tuned model—Meditron-70B [33]—exhibited paradoxically poor diagnostic performance (16.7% to 25.0% top-five accuracy across diseases and prompt conditions) substantially below that of both the proprietary benchmark and the two general-domain open-weight alternatives of comparable parameter scale. This counterintuitive result was further reinforced by the specialist-committee assessment, in which Meditron-70B generated the lowest mean number of biologically plausible hypotheses per case (1.0–1.7 of 5; Table 2) and the highest proportion of clinically impossible diagnoses among the four models. The finding is consistent with a growing body of recent evidence indicating that domain-specific fine-tuning on biomedical corpora does not automatically translate into superior clinical reasoning and that general-domain models frequently match or outperform their medically fine-tuned counterparts on clinical benchmarks [34]. Several non-mutually exclusive mechanisms may account for this pattern in the NTD context: (i) the training corpora used for medical-domain models are heavily skewed towards English-language biomedical literature and towards conditions prevalent in high-income settings, with NTD-specific content under-represented [38]; (ii) continued pre-training on narrow biomedical text may erode the broad reasoning capabilities acquired during foundational training, a phenomenon known as catastrophic forgetting; and (iii) the linguistic distance between Meditron-70B’s predominantly English training corpus and the Brazilian Portuguese clinical material used in the present study may further amplify this gap. Whatever the underlying mechanism, the implication is methodologically important: the hypothesis that medical-domain fine-tuning automatically confers diagnostic advantage cannot be assumed and must be empirically tested for each clinical context.

Third, gender-attributed persona-prompt manipulation did not produce statistically significant differences in diagnostic accuracy for any of the four models or either of the two evaluated diseases (all *p* > 0.05; Figure 2 and Section 3.3). Within-model comparisons across the two prompt conditions revealed numerical differences ranging from 0 to 16.7 percentage points, with a consistent directionality favouring the female prompt across most model–disease combinations: LLaMA 3 70B and Mixtral 8x7B showed gains of 8.3 to 16.7 percentage points under the female prompt, ChatGPT-4o showed minimal variation (0 percentage points in CD; +8.3 in VL), and Meditron-70B showed inconsistent patterns. The specialist-committee plausibility assessment exhibited the same direction of trend, with a small but consistent numerical advantage for the female prompt across all four models and both diseases (Table 2).

Two interpretations of this pattern must be carefully distinguished. First, in the present sample and under the present design, the linguistic gender marker of the persona was not a robust or reliable driver of diagnostic accuracy: the within-subject paired analysis—in which each clinical case acted as its own control across conditions—provides direct evidence that no systematic effect was detected. Second, however, the consistent directionality of the small numerical differences, combined with the limited statistical power afforded by *n* = 12 paired observations per disease, means that the present study cannot rule out the existence of small effects that would emerge with larger samples. Recent literature has documented that LLMs exhibit sensitivity to demographic and non-clinical cues embedded in patient cases and physician personas [25]. For example, Liu et al. [25] assigned gender as a persona attribute to multiple proprietary and open-source LLMs using clinical vignettes from the New England Journal of Medicine Challenge and reported substantial inconsistency across LLM gender assignments—a finding that complements our observation of persona-prompt sensitivity in a distinct clinical domain. Altering gender markers can also shift LLM clinical reasoning, occasionally leading to disparities in the recommended urgency of care [26]. Open-weight models also possess inherent statistical biases inherited from their pre-training corpora, which can alter the severity and linguistic directness of medical outputs as a function of gender markers [27].

Whatever the eventual interpretation of the small numerical trend, the present results carry a clear practical implication: in the development of clinical AI prompts for NTD differential diagnosis in Brazilian Portuguese, the gender of the attributed persona does not exert a strong systematic influence and need not be a primary concern of prompt engineering. Nevertheless, standardisation of prompts (including explicit, documented decisions about persona attributes) remains essential for reproducible and equitable AI-assisted diagnostics across studies and clinical settings.

Fourth, the specialist-committee plausibility assessment revealed that diagnostic accuracy alone is an incomplete measure of LLM clinical reasoning quality (Table 2). ChatGPT-4o not only identified the target disease more frequently but also generated differential diagnosis lists of substantially higher overall biological plausibility (mean of 4.3 to 4.7 plausible hypotheses per case across diseases and prompt conditions). In sharp contrast, Meditron-70B frequently produced clinically impossible diagnoses (mean of 1.0 to 1.7 plausible hypotheses per case, equivalent to 3.3 to 4.0 implausible hypotheses), raising substantive concerns about its safety profile in potential clinical applications. This dissociation between accuracy and plausibility carries direct clinical implications. A model could, in principle, achieve acceptable target-disease accuracy while embedding the correct diagnosis within a list of clinically implausible alternatives—a configuration that would mislead clinical decision-making, trigger unnecessary diagnostic tests, and contribute to alert fatigue among healthcare professionals [30]. This reinforces the methodological argument that LLM evaluation for clinical deployment should incorporate expert-validated qualitative assessment of every generated hypothesis, not merely binary detection of the target diagnosis—particularly in safety-critical contexts such as NTD diagnosis, where downstream confirmatory testing is, itself, resource-intensive and time-sensitive.

Fifth, these findings carry direct relevance for the broader debate around AI-assisted diagnostic deployment in resource-constrained NTD-endemic settings, where data sovereignty, hardware limitations, and offline operation are critical operational constraints [20]. Open-weight LLMs deployed locally—as was the case for LLaMA 3 70B, Meditron-70B, and Mixtral 8x7B in the present study—offer advantages in cost, regulatory compliance, and patient data confidentiality that cloud-hosted proprietary models cannot match in many Brazilian public-hospital settings. However, our results indicate that currently available 70B-parameter open-weight models exhibit substantially lower diagnostic accuracy and lower hypothesis-plausibility scores than ChatGPT-4o for both evaluated NTDs, suggesting that practical deployment in this clinical context would require either careful model selection, prompt-engineering optimisation, or—most importantly—human-in-the-loop oversight protocols designed to capitalise on model strengths while mitigating documented failure modes.

A particularly promising avenue for future deployment lies in small language models (SLMs) specifically designed for clinical reasoning. Recent SLMs in the 3- to 10-billion-parameter range have demonstrated performance competitive with much larger models on standard medical benchmarks while remaining deployable on modest institutional hardware—a critical consideration for tertiary-care services in NTD-endemic regions of Latin America, sub-Saharan Africa, and South Asia. Meerkat-7B and Meerkat-8B [39], distilled from chain-of-thought reasoning trajectories grounded in medical textbooks, have been reported to achieve performance comparable to that of substantially larger general-domain models on multiple clinical-reasoning benchmarks. Phi-4-mini [40] offers an alternative architectural approach at the 3.8-billion-parameter scale, achieving competitive performance on standard medical reasoning benchmarks while remaining deployable on modest hardware. Whether SLMs of this class, trained or fine-tuned on Brazilian Portuguese clinical material and on NTD-specific corpora, can match or surpass the performance of the 70B open-weight models evaluated in the present study constitutes, in our view, the most pressing question for the next generation of clinical AI evaluation in NTD-endemic settings.

### 4.1. Strengths

This study has several notable strengths. First, it employed real anonymised patient data rather than constructed clinical vignettes, enhancing clinical realism and mitigating the inflated accuracy often observed when LLMs are tested solely on artificial medical-examination questions [30]. Second, the paired within-subject experimental design—isolating a single linguistic variable (the gender of the attributed persona) while holding clinical content, professional credentials, task instruction, and output format strictly constant across conditions—provides rigorous evidence on prompt sensitivity, with each clinical case serving as its own control. Third, the inclusion of four LLMs spanning proprietary and open-weight categories and three distinct architectural families among the open-weight models (dense decoder, sparse mixture of experts, and medical-domain fine-tuned) broadens the generalisability of the findings beyond any single model lineage. Fourth, the independent five-member specialist-committee assessment of all generated hypotheses adds a qualitative dimension rarely incorporated in LLM evaluation studies, as visualised in Table 2, with most published reports focusing exclusively on target-disease accuracy. Fifth, the focus on two NTDs in Brazilian Portuguese addresses a critical gap, since most LLM diagnostic studies have concentrated on conditions prevalent in high-income settings and on the English language [41].

### 4.2. Limitations

Several limitations should be acknowledged. First, the sample size of 12 cases per disease provides limited statistical power for detecting small effects: the paired Wilcoxon signed-rank test used in this design has approximately 80% power to detect medium-to-large effects (Cohen’s h ≥ 0.6) with two-tailed α = 0.05 but lower power for the smaller effect sizes suggested by the numerical trends observed in the present data. Larger multi-centre samples—in our view, on the order of 50 to 100 paired cases per disease—would be necessary to formally characterise the magnitude (or definitive absence) of persona-prompt effects on diagnostic accuracy in this clinical context.

Second, the study evaluated a single prompt structure; alternative prompt-engineering strategies (such as chain-of-thought scaffolding, role-instructed examples, or retrieval-augmented prompting) may yield different relative model rankings and different sensitivities to persona attributes [29]. Third, the LLMs were evaluated between 29 July and 30 August 2024 using the model versions available at that time; in particular, ChatGPT-4o was the state-of-the-art proprietary model from OpenAI throughout the study period. Subsequent releases—GPT-5 (August 2025) and GPT-5.5 (April 2026)—postdate the present evaluation, and prospective replication using these newer models, alongside the most recent open-weight releases such as LLaMA 3.3 and LLaMA 4, will be needed to assess whether the patterns reported here generalise across model generations.

Fourth, clinical cases were drawn from a single institution (HU-UNIVASF) in a specific hyperendemic region of Brazil (the São Francisco Valley), which may limit generalisability to other geographic and clinical contexts. Fifth, the four-category committee classification of diagnostic plausibility, while informative, does not capture the full nuance of clinical reasoning quality (e.g., severity of error and ranking of plausible alternatives within a list); more granular frameworks should be developed for future evaluations. Sixth, the study was conducted exclusively in Brazilian Portuguese; cross-linguistic validation in Spanish, French, and English would help disentangle language-specific from model-specific contributions to the observed patterns of accuracy and persona-prompt sensitivity.

## 5. Conclusions

This study systematically compared one proprietary (ChatGPT-4o) and three open-weight large language models (LLaMA 3 70B, Meditron-70B, and Mixtral 8x7B) for the differential diagnosis of Chagas disease and visceral leishmaniasis using real anonymised clinical cases under a paired persona-prompt design. ChatGPT-4o consistently outperformed the three open-weight alternatives on both the top-five accuracy and biological plausibility of the generated differentials. The medically fine-tuned Meditron-70B exhibited paradoxically poor performance, challenging the assumption that medical-domain fine-tuning automatically confers a diagnostic advantage. Gender-attributed persona-prompt variation did not produce statistically significant differences in any model–disease combination, although a consistent small numerical trend favouring the female prompt warrants verification in larger samples. The independent specialist-committee assessment confirmed that accuracy alone is an incomplete measure of LLM clinical reasoning quality, supporting the inclusion of expert-validated qualitative assessment in future evaluations.

These findings lead to five concrete directions for future research: (i) larger multicentre samples (50–100 paired cases per disease) to formally characterise small persona-prompt effects; (ii) systematic inclusion of small language models such as Meerkat-7B/8B [39] and Phi-4-mini [40], which are deployable on modest institutional hardware; (iii) evaluation of additional prompt-level variables (clinician seniority, language register, and chain-of-thought scaffolding); (iv) cross-linguistic validation in Spanish, French, and English; and (v) prospective replication using GPT-5, GPT-5.5, and the latest open-weight releases. As LLMs become increasingly embedded in clinical workflows [42], establishing rigorous and reproducible evaluation standards is a precondition for their safe, equitable, and effective integration into the diagnostic care of patients with neglected tropical diseases.

## Data Availability

The data presented in this study are available upon request from the corresponding author. The data are not publicly available due to ethical restrictions.

## Supplementary Materials

The following supporting information can be downloaded at: https://www.mdpi.com/article/10.3390/healthcare14101385/s1, Box S1: English translation of the male and female gender-attributed persona prompts administered in Brazilian Portuguese to the four large language models evaluated in this study.

## Abbreviations

The following abbreviations are used in this manuscript:

AI	Artificial Intelligence
AIDS	Acquired Immunodeficiency Syndrome
CAAE	Certificate of Presentation for Ethical Appraisal
CD	Chagas Disease
CI	Confidence Interval
CNPq	Conselho Nacional de Desenvolvimento Científico e Tecnológico
EBSERH	Brazilian Hospital Services Company
EPFL	École Polytechnique Fédérale de Lausanne
FACEPE	Fundação de Amparo à Ciência e Tecnologia do Estado de Pernambuco
GPT	Generative Pre-trained Transformer
HIV	Human Immunodeficiency Virus
HU-UNIVASF	Hospital Universitário da Universidade Federal do Vale do São Francisco
LAPEDH	Human Performance Research Laboratory
LLaMA	Large Language Model Meta AI
LLM	Large Language Model
NTD	Neglected Tropical Disease
SD	Standard Deviation
UPE	University of Pernambuco
VL	Visceral Leishmaniasis
